# Clonogenic growth of human breast cancer cells co-cultured in direct contact with serum-activated fibroblasts

**DOI:** 10.1186/bcr995

**Published:** 2005-01-28

**Authors:** Michael Samoszuk, Jenny Tan, Guillaume Chorn

**Affiliations:** 1 Pathology Department, University of California, Irvine, California, USA; 2 Biology Department, Stanford University, Stanford, California, USA

**Keywords:** fibroblasts, metastatic, microarrays, myofibroblasts, serum

## Abstract

**Introduction:**

Accumulating evidence suggests that fibroblasts play a pivotal role in promoting the growth of breast cancer cells. The objective of the present study was to characterize and validate an *in vitro *model of the interaction between small numbers of human breast cancer cells and human fibroblasts.

**Methods:**

We measured the clonogenic growth of small numbers of human breast cancer cells co-cultured in direct contact with serum-activated, normal human fibroblasts. Using DNA microarrays, we also characterized the gene expression profile of the serum-activated fibroblasts. In order to validate the *in vivo *relevance of our experiments, we then analyzed clinical samples of metastatic breast cancer for the presence of myofibroblasts expressing α-smooth muscle actin.

**Results:**

Clonogenic growth of human breast cancer cells obtained directly from *in situ *and invasive tumors was dramatically and consistently enhanced when the tumor cells were co-cultured in direct contact with serum-activated fibroblasts. This effect was abolished when the cells were co-cultured in transwells separated by permeable inserts. The fibroblasts in our experimental model exhibited a gene expression signature characteristic of 'serum response' (i.e. myofibroblasts). Immunostaining of human samples of metastatic breast cancer tissue confirmed that myofibroblasts are in direct contact with breast cancer cells.

**Conclusion:**

Serum-activated fibroblasts promote the clonogenic growth of human breast cancer cells *in vitro *through a mechanism that involves direct physical contact between the cells. This model shares many important molecular and phenotypic similarities with the fibroblasts that are naturally found in breast cancers.

## Introduction

There is now increasing evidence that stromal cells play a pivotal role in promoting the growth of most carcinomas, including breast cancer [1-4]. The myofibroblast is the predominant type of stromal cell found in most carcinomas [1,5,6]. Myofibroblasts are defined by their characteristic expression of α-smooth muscle actin as well as certain other markers such as vimentin and desmin [1]. Tumor-associated myofibroblasts are believed to originate from normal fibroblasts and are similar or identical to the myofibroblasts found in healing wounds [1]. They are largely responsible for the desmoplasia that is characteristically present in carcinomas because they secrete large amounts of collagen and other extracellular matrix proteins.

A large body of work has been done to investigate the interactions between fibroblasts and carcinoma cells [1-4,7-9]. Recently, an orthotopic xenograft model was created in mice in which both the stromal and epithelial components of the reconstructed mammary gland are of human origin [10]. This complex model again underscored the critical role played by heterotypic interactions in human breast carcinogenesis. Many other *in vitro *and *in vivo *studies have demonstrated that fibroblasts can promote the growth of cancer cells [11-16]. We are not aware, however, of any previous reports of studies investigating the ability of fibroblasts to promote the clonogenic growth of small numbers of primary breast cancer cells *in vitro*.

**Figure 1:**
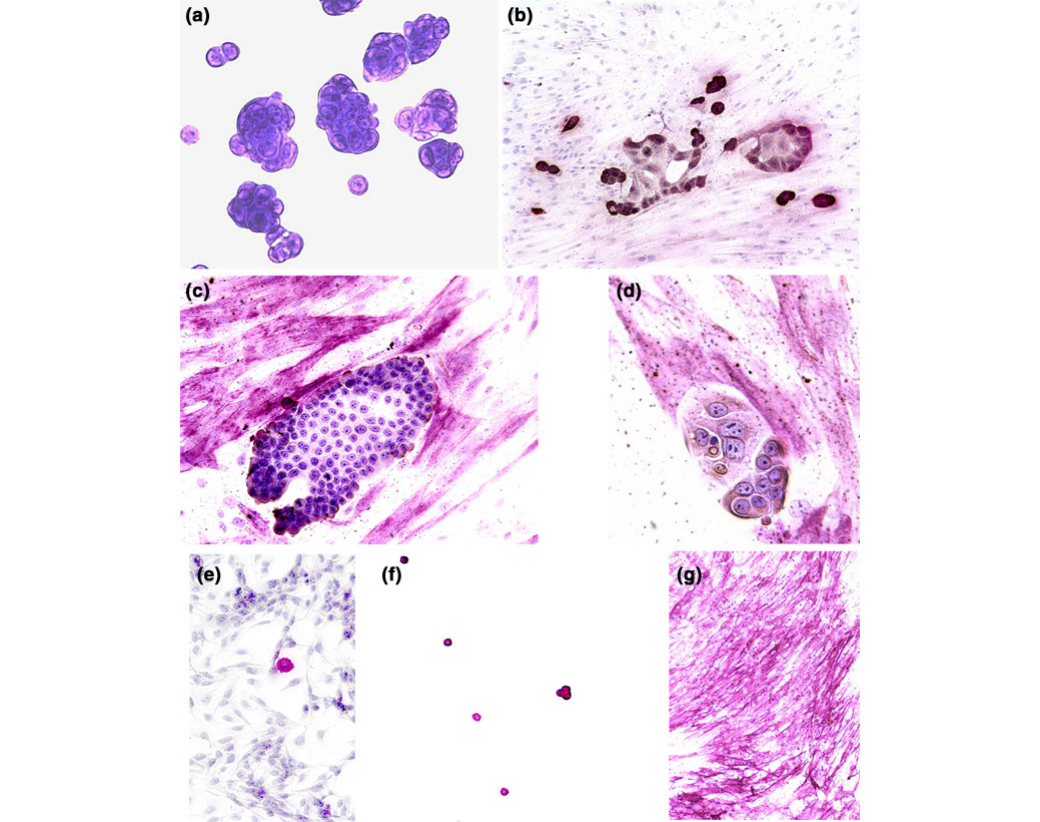
Microscopic appearance of tumor cells and fibroblasts. **(a) **Numerous clumps of UACC-812 tumor cells resembling abortive mammary glands are evident. **(b) **UACC-812 breast cancer cells seeded at low density on a monolayer of serum-activated fibroblasts. Note the gland formation and the invasive infiltration by tumor cells into fibroblasts. Primary tumor cells derived from **(c) **human ductal carcinoma *in situ *and from **(d) **invasive ductal carcinoma co-cultured with fibroblasts yielded clonogenic growth next to myofibroblasts that expressed α-smooth muscle actin (pink immunostain). Breast cancer cells grown **(e) **on a monolayer of B16 cells or **(f) **without a monolayer of fibroblasts generally grew as individual cells identifiable by their red staining for epithelial membrane antigen. **(g) **Serum-activated fibroblasts also uniformly expressed abundant syndecan-1, as detected by immunocytochemical staining.

To address this issue, we first co-cultured low numbers of a slow growing human breast cancer cell line (UACC-812) with a monolayer of normal human fibroblasts derived from human breast tissue. We selected this tumor cell line for our initial experiments because it was derived in 1991 from a ductal carcinoma (stage II, grade IV) and closely mimics the glandular morphology (Fig. 1a) and slow growth rate (doubling time > 100 hours) of primary breast cancer [17]. In order to validate the potential clinical relevance of our experiments, we then co-cultured a monolayer of serum-activated fibroblasts with tumor cells obtained directly from human breast cancer samples. Gene expression profiling by microarrays and immunocytochemistry were used to characterize the serum-activated fibroblasts. Finally, we examined 25 randomly selected surgical specimens containing metastatic breast cancer of varying grades for the presence of fibroblasts expressing α-smooth muscle actin in close proximity to tumor cells. Here, we report the novel observation that serum-activated fibroblasts promote the clonogenic growth of small numbers of breast cancer cells obtained directly from human tumor samples. Moreover, we show that fibroblasts activated by serum *in vitro *share many molecular features with the fibroblasts that are naturally and abundantly present within primary human breast cancers and metastases.

## Methods

### Breast cancer cell line and fibroblasts

The UACC-812 human breast cancer cell line (American Type Culture Collection [ATCC], Manassas, VA, USA) was passaged in Leibovitz's medium supplemented with 15% fetal calf serum prior to use. Normal fibroblasts (CCD-1068SK; ATCC) obtained from the breast of a 65-year-old female were passaged at 37°C in minimal essential medium (Eagle's) supplemented with 2 mmol/l L-glutamine, Earle's balanced salt solution (1.5 g/l), sodium bicarbonate, 0.1 mmol/l nonessential amino acids, 1 ml sodium pyruvate, and 10% fetal calf serum in a 5% carbon dioxide atmosphere. All cell culture reagents were obtained from ATCC. Our co-culture experiments utilized confluent monolayers of fibroblasts that had been passaged no more than 14 days. This precaution ensured that the fibroblasts were not senescent or transformed.

### Immunocytochemical assays of cultured cells

The presence of α-smooth muscle actin, epithelial membrane antigen (a marker of breast cancer cells), and syndecan-1 was detected by *in situ *immunostaining of methanol-fixed cells using prediluted murine monoclonal antibodies directed against α-smooth muscle actin, or syndecan-1, or epithelial membrane antigen (Chemicon International, Temecula, CA, USA). Bound antibody was then detected with an alkaline phosphatase procedure (IHC Select^® ^AP/Fast Red; Chemicon International) in accordance with the manufacturer's directions.

### Clonogenic co-culture assay

We seeded 100 UACC-812 breast cancer cells into individual wells of a 96-well cell culture plate containing a confluent monolayer of fibroblasts growing in fibroblast growth medium supplemented with 10% fetal calf serum. Controls included co-culture with fibroblasts in serum-free medium, co-culture with a monolayer of a nonfibroblast cell line (murine B16 melanoma), and culture in medium with fetal calf serum but without fibroblasts. In order to determine whether a soluble factor was responsible for any effect that we observed, we also co-cultured the fibroblasts and breast cancer cells separately on opposite sides of permeable transwell inserts with a 0.2 μm pore size (Nalge-Nunc International, Rochester, NY, USA). The inserts permitted diffusion of any soluble growth factors produced by the fibroblasts but prevented direct contact between the cells. All of the experimental and control combinations were performed in triplicate wells of 96-well culture plates.

At intervals of 3–4 days, fresh medium was added. After 14 days the cells were fixed with 70% ethanol for 10 min prior to staining for 3 min with 0.1% toluidine blue. Using inverted microscopy, we counted the number of colonies containing 12 or more tumor cells in each well. A colony was defined as a cluster of epithelial cells in direct contact with one another. The means and standard deviations for each condition were compared statistically using pair-wise comparisons with the appropriate control in an unpaired, two-tailed t-test.

### Proliferation and apoptosis assays

In order to determine whether serum-activated fibroblasts promote the growth of breast cancer cells *in vitro *even at higher densities of tumor cells, we used flow cytometry to characterize the apoptotic and proliferative rates of breast cancer cells co-cultured with fibroblasts. Confluent monolayers of fibroblasts in T-75 culture flasks (Corning Inc., Corning, NY, USA) were seeded with 500,000 breast cancer cells and co-cultured for 1 week with a medium change at 4 days. On day 7 we separated the breast cancer cells from the fibroblasts using magnetic beads conjugated to epithelial membrane antigen (DYNAL MPC-S Kit; Dynal AS, Oslo, Norway) in accordance with the manufacturer's directions. The negative control consisted of 500,000 breast cancer cells cultured in the same medium but without fibroblasts.

The purified breast cancer cells were then analyzed for their proportions of proliferating and apoptotic cells using the Absolute-S™ Kit for Measuring Cell Proliferation by Dual Color Flow Cytometry (Chemicon International) in accordance with the manufacturer's directions. The stained cells were analyzed on a Coulter^® ^Epics^®^-MCL (Coulter, Hialeah, FL, USA) flow cytometer using appropriate positive and negative controls.

### Co-culture of primary breast cancer cells and fibroblasts

We aseptically dissected small pieces of tissue (2–3 mm in diameter) from three cases of invasive ductal carcinoma of the breast, three cases of ductal carcinoma *in situ*, and seven specimens of normal breast tissue. The tissue fragments were then mechanically disaggregated using Medicons™ (BD Biosciences, San Jose, CA, USA) with a 50 μm mesh size. This method routinely yielded between 1000 and 10,000 viable epithelial cells, as determined by trypan blue staining and expression of epithelial membrane antigen.

We seeded about 100 epithelial cells into individual wells of 96-well cell culture plates containing a monolayer of fibroblasts. The control consisted of epithelial cells seeded into wells without fibroblasts. At the end of 14 days of co-culture, the cells were stained *in situ *with toluidine blue as described above, and the colonies of tumor cells (defined as 12 or more adjacent epithelial cells) in each well were counted using inverted microscopy.

### Target preparation/processing for GeneChip analysis

We used gene expression analysis on Affymetrix U133A cDNA microarrays (Affymetrix Inc., Santa Clara, CA, USA) to compare the molecular signatures of serum-activated fibroblasts with those of serum-starved fibroblasts (experiment 1). We also compared the gene expression patterns of serum-activated fibroblasts cultured alone with those of serum-activated fibroblasts co-cultured with breast cancer cells for 3 days before being purified with magnetic beads (experiment 2). All assays were performed in duplicate at the UCI DNA Microarray Core Facility. Total RNA isolated from separate cultures of serum-activated, serum-starved, and purified, co-cultured fibroblasts was processed as recommended by Affymetrix Inc. [18]. The gene expression results were quantified and analyzed using GCOS 1.1.1 software (Affymetrix Inc.) and/or ArrayAssist's gcRMA (Iobion Informatics LLC, LaJolla, CA, USA) using default values. Gene expression in a sample was considered to be increased if the signal intensity was interpreted to be positive and at least fourfold higher (with *P *< 0.001) than the baseline (defined as serum-starved fibroblasts in experiment 1 or serum-activated fibroblasts cultured alone in experiment 2).

### *In vivo *metastasis study

In order to determine whether fibroblasts expressing α-smooth muscle actin are in direct physical contact with metastases of breast cancer cells, we immunostained 25 randomly selected specimens of metastatic human breast cancer (ductal adenocarcinoma type) from the surgical pathology and autopsy archives at UCI Medical Center (Orange, CA, USA). These included four samples of well differentiated ductal adenocarcinoma, nine samples of moderately differentiated adenocarcinoma, and 12 samples of poorly differentiated carcinoma. Ten of the metastases were to lymph nodes, six were to bone marrow, five were to liver, two were to lung, one was to brain, and one was to pleura. The age range of the patients was 42–69 years. The study set included eight samples with micrometastases, which we defined as fewer than 100 visible tumor cells.

The slides were stained with prediluted murine monoclonal antibody directed against α-smooth muscle actin (Ventana Medical Systems, Phoenix, AZ, USA). Bound antibody was detected using a biotin-streptavidin procedure employing horseradish peroxidase as the detecting enzyme. Appropriate positive and negative controls were included in each staining run. In order to confirm that cells expressing smooth muscle actin were myofibroblasts and not myoepithelial cells, we also stained serial sections of the same tissues with antibodies directed against CD10 and smooth muscle myosin heavy chain. These markers are preferentially expressed on myoepithelial cells [19] rather than myofibroblasts.

## Results

### Microscopic appearance of co-cultured cells

When co-cultured with a monolayer of fibroblasts, the UACC-812 cell line consistently produced colonies of tumor cells that irregularly infiltrated the fibroblasts (Fig. 1b). Significantly, after just 14 days in co-culture the primary tumor cells derived directly from clinical specimens of ductal carcinoma *in situ *(Fig. 1c) or invasive ductal carcinoma (Fig. 1d) also produced multiple colonies in close proximity to fibroblasts. The epithelial cells in these colonies exhibited the usual cytologic features of malignancy, including prominent nucleoli, a high nuclear:cyoplasmic ratio, and marked cytologic atypia. Some of these colonies consisted of nearly 100 tumor cells (Fig. 1c), suggesting a doubling time of only 12 hours. It was also noteworthy that the fibroblasts adjacent to the colonies of tumor cells invariably expressed abundant α-smooth muscle actin (pink-staining fibroblasts in Fig. 1c,d). When the UACC-812 cell line was co-cultured on a monolayer of B16 melanoma cells, clonogenic growth was nearly absent (Fig. 1e). Moreover, UACC-812 cells cultured in the absence of fibroblasts survived only as individual cells or in very small colonies, generally of fewer than eight cells (Fig. 1f). The serum-activated fibroblasts in this model system uniformly expressed syndecan-1 (Fig. 1g). There was no evidence of overgrowth by fibroblasts during the course of the experiment.

### Clonogenic growth of breast cancer cells is promoted by direct contact with serum-activated fibroblasts

**Figure 2:**
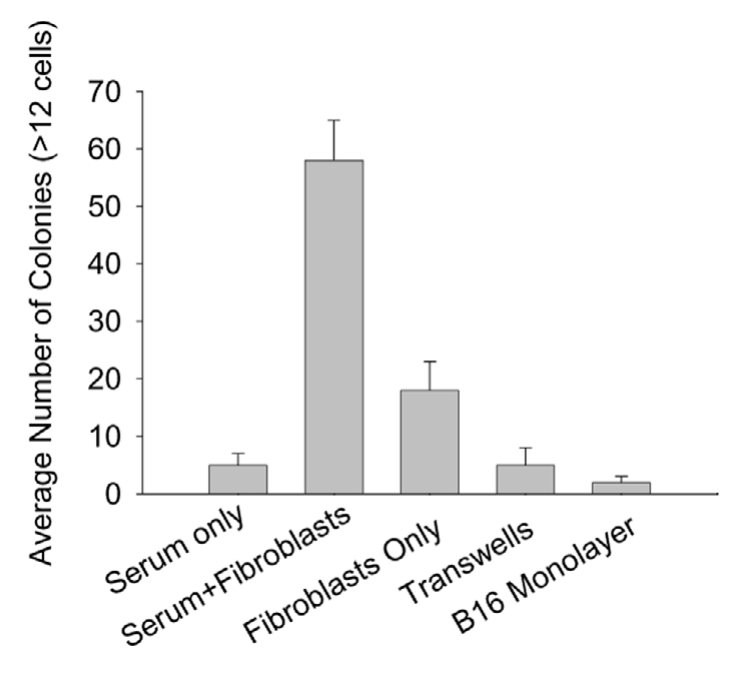
Results of the co-culture assay. Each bar represents the mean and standard deviation of triplicate measurements. Tumor cells seeded on a monolayer of serum-activated fibroblasts produced the highest average number of colonies. There were significantly fewer colonies if fibroblasts or serum were omitted. Co-culture with fibroblasts separated by permeable transwell inserts or on a monolayer of B16 cells yielded low numbers of colonies that were statistically indistinguishable from the control using tumor cells grown in serum-supplemented medium without fibroblasts.

We performed co-culture experiments under a variety of conditions that are summarized in Fig. 2. Tumor cells co-cultured with serum-activated fibroblasts produced significantly (*P *< 0.01) more colonies than did tumor cells cultured in serum-containing medium without fibroblasts or with fibroblasts in serum-free medium. Co-culture of the tumor cells and fibroblasts in separate chambers of transwells yielded low numbers of colonies, statistically identical to culture of tumor cells alone, as did co-culture on a monolayer of B16 cells. Fibroblast conditioned medium had no detectable effect on clonogenic growth of tumor cells grown in the absence of fibroblasts (data not shown).

### Serum-activated fibroblasts promote proliferation of breast cancer cells even at high densities of tumor cells

**Figure 3:**
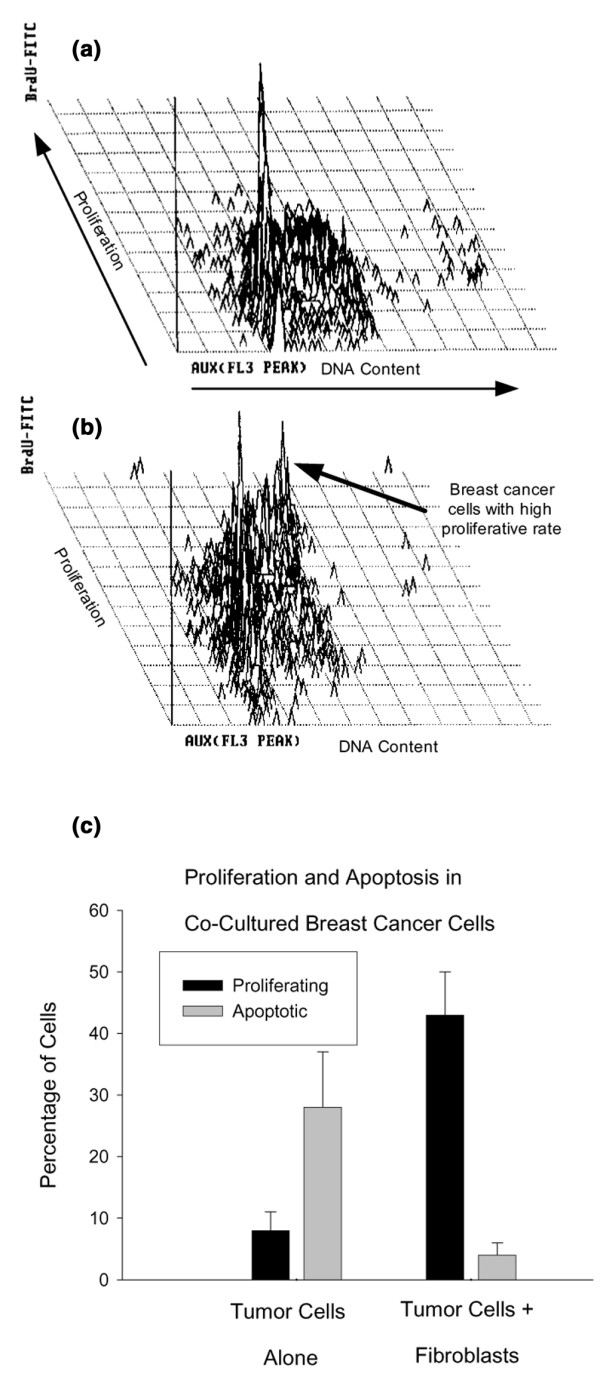
Results of flow cytometric measurements of proliferation and apoptosis. There was a distinct peak of **(b) **breast cancer cells with a high proliferative rate compared with **(a) **breast cancer cells cultured without fibroblasts. **(c) **Tumor cells grown on fibroblasts had a significantly higher proliferative rate and a lower apoptotic rate than did tumor cells cultured alone.

A graphical summary of the proliferation and apotosis data from triplicate flow cytometry assays is presented in Fig. 3. These data confirmed that high densities of breast cancer cells co-cultured with fibroblasts had significantly higher proliferative rates and lower apoptotic rates than did breast cancer cells cultured in serum-supplemented medium without fibroblasts.

### Clonogenic growth of primary tumor cells derived from clinical samples

The results of these experiments are shown in Table 1. Each number in the table represents the total number of colonies of tumor cells obtained when the epithelial cells from one sample were cultured in the presence or absence of serum-activated fibroblasts. Notably, all of the clinical samples of ductal carcinoma *in situ *and invasive carcinoma yielded substantially greater numbers of colonies when the epithelial cells were co-cultured with fibroblasts. In addition, the average size of the colonies produced by carcinoma cells co-cultured with fibroblasts (average 18 cells/colony) appeared to be substantially greater than the colonies produced by tumor cells cultured in the absence of fibroblasts (average eight cells/colony). The sample of invasive carcinoma that produced 33 colonies in the absence of exogenous, serum-activated fibroblasts was notable for the presence of numerous fibroblasts in the wells of the negative control, thereby suggesting contamination of the primary cancer cells by tumor-associated fibroblasts in that sample. The other clinical samples had no visible contaminating fibroblasts in the wells that were seeded with tumor cells alone. There was only minimal clonogenic growth of cells obtained from benign breast tissues, and these cells appeared cytologically benign.

### Gene expression profile of serum-activated fibroblasts

Because the gene expression profile of tumor-associated fibroblasts *in situ *has previously been described [20-22], we used cDNA microarrays to compare the gene expression patterns of the serum-activated fibroblasts used in our experiments with serum-starved fibroblasts, and to compare serum-activated fibroblasts with serum-activated fibroblasts that were co-cultured with tumor cells.

The results, shown in Table 2, confirm that serum-activated fibroblasts differentially upregulated genes related to myofibroblast differentiation (*LOXL2*, *PLOD2*, *PLAUR*, *TAGLN*, *TPM2*, *MYL6*, *TFPI2*) relative to serum-starved fibroblasts. In addition, there was upregulated expression of two genes that have previously been shown [20,22] to be highly associated with tumor-associated fibroblasts in breast cancer (*CXCL12 *and *CLIC4*) and upregulation of the *COX2 *gene. Finally, we noted that serum-activated fibroblasts also upregulated genes related to activation and function of transforming growth factor (TGF)-β (*TGFB1*, *LTBP2*, *SMAD3*). The gene expression profile of serum-activated fibroblasts co-cultured with breast cancer cells was essentially the same as the profile of serum-activated fibroblasts cultured without tumor cells (data not shown).

### α-Smooth muscle actin-positive fibroblasts are in direct contact with tumor cells in metastases of breast cancer

**Figure 4:**
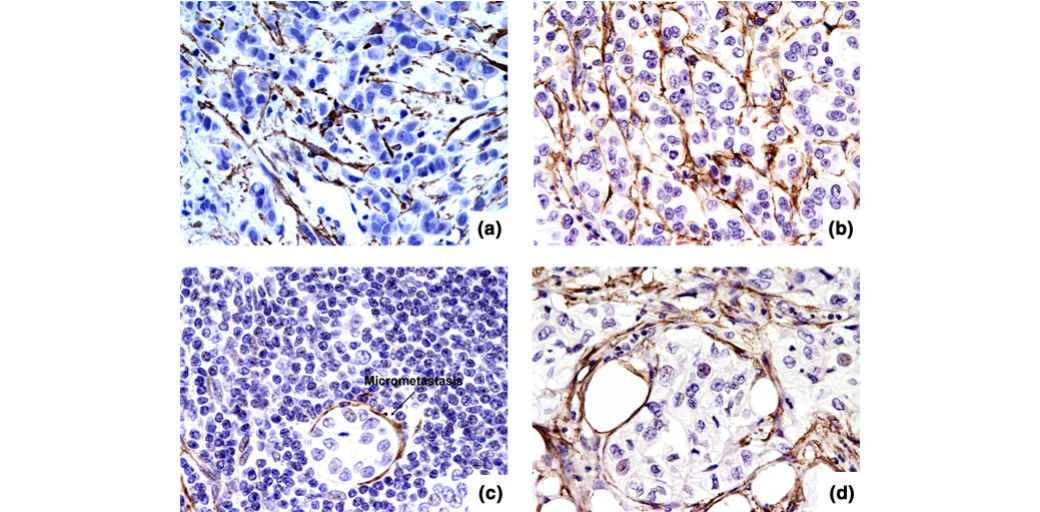
Immunohistochemical staining for α-smooth muscle actin in metastases of human breast cancer to **(a) **bone marrow, **(b) **liver, **(c) **lymph node, and **(d) **pleura. In all of the cases that we examined, there was extensive infiltration of the tumor metastases by myofibroblasts (brown stain). Note that the myofibroblasts appeared to be in direct contact with nearly all of the tumor cells in these sections. Even very early micrometastases (defined as fewer than 100 tumor cells) of well differentiated tumors to lymph nodes (panel c) and larger metastases of poorly differentiated tumors (panel d) were in very close proximity to myofibroblasts. Serial sections of the same tissues did not stain for CD10 or smooth muscle myosin heavy chain (not shown), thereby confirming that the cells are myofibroblasts rather than myoepithelial cells. Hematoxylin counter-stain was used.

There was intense staining for α-smooth muscle actin in all of the breast cancer metastases that we examined (Fig. 4). Significantly, the fibroblasts in these tissues closely enveloped the nests of tumor cells, even in the earliest micrometastases to lymph nodes (Fig. 4c). Our immunostaining results demonstrated that virtually all of the tumor cells were in direct contact or very close proximity to myofibroblasts, regardless of tumor grade or metastatic site. There was little or no staining of these same tissues with antibodies directed against CD10 or smooth muscle myosin heavy chain, thereby indicating that the cells expressing smooth muscle actin were myofibroblasts rather than myoepithelial cells.

## Discussion

We demonstrated that serum-activated human fibroblasts significantly enhance the clonogenic growth of small numbers of primary breast cancer cells obtained directly from clinical specimens. This effect requires direct physical contact between tumor cells and the serum-activated fibroblasts and cannot be replicated by fibroblast-conditioned medium, co-culture in transwells, or co-culture on a monolayer of another cell type (B16 cells). Moreover, we showed that serum-activated fibroblasts exhibit many molecular similarities to the fibroblasts that naturally infiltrate human breast cancer, including expression of syndecan-1, α-smooth muscle actin, and genes related to myofibroblast differentiation and activation by TGF-β. Finally, we demonstrated that fibroblasts expressing α-smooth muscle actin are ubiquitously present in metastases of human breast cancer and appear to be in close proximity or direct physical contact with nearly all of the tumor cells. On aggregate, these results provide further evidence of the essential role played by serum-activated fibroblasts in promoting the clonogenic growth of small numbers of breast cancer cells, both *in vitro *and *in vivo *within metastases.

It has long been known that feeder cell layers of fibroblasts can promote the growth of various cell lines in culture. Surprisingly few studies, however, have directly related this effect of feeder cells to the clonogenic growth of small numbers of primary breast cancer cells *in vitro*. In one such study investigators separated human breast cancer cell lines from various types of fibroblasts with a microporous membrane [7]. Under these conditions, tumor-associated fibroblasts did not influence the proliferation of the two breast cancer cell lines that were tested. This finding is consistent with our observation that fibroblast conditioned medium also does not promote the survival or clonogenic growth of small numbers of breast cancer cells, and suggests that a soluble growth factor or mediator is not involved in the growth enhancement effect produced by fibroblast feeder cell layers.

In a later study, Brooks and coworkers [8] demonstrated that tumor-derived epithelial cells grew significantly better on stroma produced by mammary fibroblasts than on bone marrow stroma. Although it is generally difficult to culture malignant epithelial cells from primary human breast cancers, those investigators noted that a large proportion of their samples (78%) successfully grew when co-cultured with fibroblasts. They attributed this observation to their source of tumor cells (metastases to lymph nodes), but an equally plausible explanation is that the stromal cell layers that they used in their experiments promoted the growth of small numbers of tumor cells. Our study significantly extends the findings of Brooks and coworkers [8] by demonstrating that the ability of serum-activated fibroblasts to enhance the clonogenic growth of small numbers of breast cancer cells *in vitro *is not limited to cancer cells that have metastasized to lymph nodes.

Although serum-activated fibroblasts promoted clonogenic growth of breast cancer cells in our experimental model, they did not appear to have a similar effect on benign breast epithelial cells. Specifically, cells derived from benign breast tissues yielded only small colonies of cells with benign cytologic features as compared with cells derived from malignant tissues. We attribute the slow growth of the benign epithelial cells from the normal breast samples to our use of a relatively un-enriched cell culture medium that had been optimized for growth of normal fibroblasts rather than epithelial cells. Benign mammary epithelial cells normally require more specialized additives such as epidermal growth factor and bovine pituitary extract for optimal growth. Hence, our clonogenic and cytologic findings clearly suggest that serum-activated fibroblasts have a more potent growth-promoting effect on malignant epithelial cells than on benign breast cells.

Therefore, an important practical implication of our findings is that the well known difficulty in growing primary human breast cancer cells *in vitro *might be alleviated by simply co-culturing the tumor cells with a monolayer of serum-activated, nonsenescent and nontransformed fibroblasts. In this regard, it is interesting to note that virtually all current methods for growing primary human breast cancer cells rely on culturing purified epithelial cells that have been separated from the fibroblasts [10,11] in order to prevent overgrowth by fibroblasts. Our data indicate that a monolayer of serum-activated fibroblasts provides an excellent substrate for growing small numbers of primary, human breast cancer cells *in vitro *without overgrowth by fibroblasts. In our model, the normal, nonsenescent fibroblasts merely formed a monolayer and then appeared to stop growing. We attribute this property to our use of normal, nonsenescent fibroblasts that exhibited contact inhibition of growth, which is characteristic of nontransformed cells.

Our observations also raise several important questions. For example, how does serum activate fibroblasts? To what extent do serum-activated fibroblasts *in vitro *resemble their *in vivo *counterparts in human breast cancer? By what mechanism(s) do serum-activated fibroblasts promote the clonogenic growth of breast cancer cells?

Serum – the fluid component of clotted blood – contains abundant coagulation-related substances, including thrombin, TGF-β released from degranulating platelets, and other serine proteases. The data from our gene expression studies raise the intriguing possibility that TGF-β released from degranulating platelets into clotting blood might be involved in activating the fibroblasts. Specifically, we observed high levels of expression of a chloride intracellular channel gene, *CLIC4*, that is differentially expressed in fibroblasts converted to myofibroblasts by TGF-β [20]. Morover, we observed that serum-activated fibroblasts expressed high levels of genes encoding induced TGF-β and latent TGF-β binding protein, which are expressed at high levels in cultured human myofibroblasts [23]. Inasmuch as TGF-β has previously been shown to be a powerful activator of fibroblasts, our experimental observations lead us to speculate that TGF-β released from degranulating platelets into serum could play a major role in the activation of tumor-associated fibroblasts by serum.

Experiments using DNA microarrays have demonstrated that fibroblasts exhibit a temporal program of gene expression in response to serum exposure [24]. Notably, many features of the transcriptional program appeared to be related to the physiology of normal wound repair, including the acquisition of a myofibroblast expression profile [24]. In subsequent studies the gene expression signature of the fibroblast serum response was also found in tumors and in healing wounds [21], possibly because both processes involve localized blood clotting and production of serum.

Our observations are consistent with and significantly extend these previous findings. Specifically, we showed that serum-activated fibroblasts express multiple genes that are characteristic of tumor-associated myofibroblasts, including the gene encoding CXC chemokine ligand-12, which was recently shown to be highly overexpressed in myofibroblasts associated with breast cancer [22]. In addition, we report the expression of genes related to TGF-β by serum-activated fibroblasts. This finding is important because a number of previous studies have demonstrated that primary breast cancer fibroblasts secrete abundant TGF-β [25,26]. Finally, our immunocytochemical studies demonstrated that serum-activated fibroblasts uniformly express high levels of syndecan-1, a proteoglycan that is abundantly and specifically expressed by the stromal cells and myofibroblasts that are present in the connective tissue of human breast cancer [27,28]. Hence, we conclude that the serum-activated fibroblasts used in our co-culture model have many important similarities to the tumor-associated myofibroblasts that are naturally found in breast cancer [13].

Interestingly, our gene expression data also suggest that serum may play a more important role than contact with tumor cells in activating fibroblasts because the gene expression profile of serum-activated fibroblasts co-cultured with tumor cells was essentially the same as the gene expression profile of serum-activated fibroblasts cultured alone. For example, the genes encoding cyclo-oxygenase-2 and myofibroblast-related proteins were upregulated relative to serum-starved fibroblasts in serum activated fibroblasts, regardless of whether the fibroblasts were co-cultured with breast cancer cells. This finding is consistent with the recent report of upregulation of the *COX2 *gene in stromal fibroblasts co-cultured with pancreatic cells in a transwell cell culture system [29]. In that report, however, the authors attributed the *COX2 *upregulation to a soluble factor produced by the pancreatic tumor cells. Our results clearly indicate that serum alone can also upregulate *COX2 *gene expression.

In our study we did not directly explore the mechanism by which serum-activated fibroblasts promote the clonogenic growth of breast cancer cells. Elenbaas and Weinberg [1] suggested that tumor-associated fibroblasts may promote the growth of cancer cells through production of extracellular matrix proteins and growth factors. A recent study conducted by Palmieri and coworkers [30] indicated that fibroblast growth factor 7 (keratinocyte growth factor) secreted by breast fibroblasts is a potent paracrine growth factor for human breast epithelial cells. The data from our transwell experiments, however, suggest that soluble growth factors by themselves are unlikely to promote clonogenic growth of human breast cancer. Instead, direct physical contact between fibroblasts and tumor cells appears to be necessary.

A reasonable mechanism to account for the necessity of direct physical contact was recently proposed by Maeda and coworkers [31], who showed that syndecan-1 expression by mouse embryonic fibroblasts promotes proliferation of human breast cancer cell lines. Because the heparan sulfate proteoglycans of syndecan-1 are known to facilitate the assembly of signaling complexes between growth factors and their cognate receptors on breast cancer cells [32,33], Maeda and coworkers concluded that stromal syndecan-1 on the surface of fibroblasts is a crucial factor regulating epithelial–stromal interactions in breast cancer. This model would explain the observation that syndecan-1 is required for Wnt-1-induced mammary tumorigenesis in mice [33] and would also account for our experimental findings.

## Conclusion

Our observations establish serum-activated fibroblasts as a critical promoter of clonogenic growth of small numbers of human breast cancer cells *in vitro *and suggest that similar activity might also be present in metastases of breast cancer *in vivo*. Further analysis of this interaction may lead to the development of novel therapeutic targets.

## Abbreviation

TGF = transforming growth factor.

## Competing interests

The author(s) declare that they have no competing interests.

## Authors' contributions

MKS conceived the study and drafted the manuscript. JT designed and performed the co-culture experiments. GC and JT jointly performed and analyzed the DNA microarray (gene expression) studies.

## Figures and Tables

**Table 1:** Numbers of colonies of breast cancer cells cultured with or without fibroblasts

Ductal carcinoma *in situ *(*n *= 3)		Invasive carcinoma (*n *= 3)	
With fibroblasts	Without fibroblasts	With fibroblasts	Without fibroblasts

6	2	1	0
1	0	130	33
65	12	21	0

**Table 2:** Myofibroblast-related genes that are differentially upregulated in fibroblasts activated by serum compared with serum-starved fibroblasts

Gene symbol	Gene name	Fold increase in signal intensity in serum-activated fibroblasts versus serum depleted fibroblasts (*P *< 0.001)
*LOXL2*	Lysyl oxidase-like 2	8.2
*PLOD2*	Procollagen-lysine, 2 oxoglutarate 5-dioxygenase	16
*PLAUR*	Plasminogen activator urokinase receptor	5.3
*TAGLN*	Transgelin	6.0
*TPM2*	Tropomysin 2 (beta)	4.6
*MYL6*	Myosin, light polypeptide 6, alkaline, smooth muscle	5.7
*TFPI2*	Tissue factor pathway inhibitor 2	4.8
*SMAD3*	Mothers against decapentaplegic homolog 3 (*Drosophila*)	4.0
*CXCL12*	Chemokine (C-X-C motif) ligand 12	4.5
*TGFB1*	TGF-β, induced	26.5
*LTBP2*	Latent TGF-β binding protein 2	30.2
*COX2*	Cyclo-oxygenase-2	16.4
*CLIC4*	Chloride intracellular channel 4	32

TGF, transforming growth factor.
